# Impact of Ovarian Yield—Number of Total and Mature Oocytes Per Antral Follicular Count—On Live Birth Occurrence After IVF Treatment

**DOI:** 10.3389/fmed.2021.702010

**Published:** 2021-08-24

**Authors:** Marine Poulain, Rodine Younes, Paul Pirtea, Julie Trichereau, Dominique de Ziegler, Achraf Benammar, Jean Marc Ayoubi

**Affiliations:** ^1^Gynecology Obstetric and Reproductive Medicine Department, Foch Hospital, Suresnes, France; ^2^Université Paris-Saclay, UVSQ, INRAE, BREED, Jouy-en-Josas, France; ^3^Ecole Nationale Vétérinaire d'Alfort, BREED, Maisons-Alfort, France; ^4^Biometry and Data Units of the Clinical Research Department of Foch Hospital, Suresnes, France

**Keywords:** ovarian yield, AFC, AMH, follicular output rate, IVF outcome, oocyte index

## Abstract

To assess the relation between oocytes yield including total retrieved oocytes (O)c and total mature oocytes (MII) relative to the antral follicular count (AFC) (3–9 mm in diameter) and relative to anti-müllerian hormone (AMH) ng/mL level: Oc/AFC, MII/AFC, Oc/AMH, and MII/AMH, respectively, and ART outcomes. We included retrospectively 264 IVF cycles after the first embryo transfer (ET) and after the cumulative ET (CET). The implantation rate (IR) and the live birth rate (LBR) after first ET were 31 ± 39% and 32.6%, respectively, and after CET 35 ± 38% and 45.1%, respectively. There was a significantly higher average of Oc/AFC and MII/AFC when live birth (LB) occurred after the first ET (0.82 ± 0.4 vs. 0.71 ± 0.35 and 0.57 ± 0.4 vs. 0.68 ± 0.3, respectively, *P* < 0.05). We reported a significantly higher average of MII/AFC when LB occurred after CET (0.66 ± 0.3 vs. 0.56 ± 0.30, *P* = 0.02) in comparison to the group where no LB was obtained. Increased Oc/AFC and MII/AFC ratios were associated with the occurrence of LB and increased IR after first ET (*P* < 0.05). Increased MII/AFC ratio was associated with the occurrence of LB and IR after CET (*P* = 0.02 and *P* = 0.04, respectively). After age-adjusted multivariate analyses, all these trends were confirmed (*P* < 0.05) except for the effect of MII/AFC ratio on IR after CET. In conclusion, Oc/AMH and MII/AMH ratios have no effect on the occurrence of LBR or on IR after first ET or CET at either age grouping. Ratios Oc/AFC and MII/AFC seem promising indicators to assess ovarian response.

## Introduction

ART treatments are based on ovarian stimulation (OS), for which the use of gonadotropins remains essential. Despite years of experience, the choice of the optimal dose of gonadotropins varies considerably depending on physicians' experience and the protocols established by each center. The main objective of OS is to induce multiple ovulation in order to retrieve several mature oocyte and obtain several embryos available for transfer, thus increasing ART efficiency (1–3).

In current practice, physicians adjust OS parameters by assessing ovarian reserve, including an antral follicular count (AFC), a measure of serum anti-müllerian hormone (AMH) level and baseline (day-3) serum follicle-stimulating hormone level before starting OS (4). Although all of these tests provide information to anticipate response to OS, the AMH level is the most widely used in routine practice (5–7).

Retrospective analysis of a cycle of ovarian stimulation makes it possible to evaluate the ovarian response of each patient, even if this response may vary from one cycle to another. This is why some authors describe several categories of patients according to their response to ovarian stimulation: poor responders if they obtained <3 oocytes at oocyte retrieval, suboptimal responders between 4 and 9 oocytes, normo-responders between 10 and 15 oocytes and hyper-responders for those who obtain more than 15 oocytes (8, 9).

Currently, two main parameters are recognized to predict response to ovarian stimulation by many authors: (i) plasma AMH level (10, 11) and (ii) AFC (12, 13). However, in practice, the predictive value of these two parameters may have limitations and the ovarian response may not correspond to the expected response. Therefore, the ability of these two ovarian markers to reflect oocyte quality and competence is still debated and controversial (4, 12, 13).

As a result, it is important and timely to provide simple, reproducible ovarian response analysis tools that can predict the chances of success of ART treatment.

Some authors suggested that the ovarian yield—antral follicle responsiveness to follicle-stimulating hormone administration—could be a good standardized tool for evaluating the response to stimulation and predicting ART outcome because it would take into account the baseline ovarian reserve and the final result of OS and therefore the actual ovarian potential of each patient (14). In the original study, the ovarian yield identified as the follicular output rate (FORT) index was correlated with pregnancy rates (15, 16). The FORT index was calculated as the number of pre-ovulatory follicles (16–22 mm the day of hCG injection) over the number of antral follicle (3–9 mm) at the beginning of OS. Some authors have correlated the FORT index with oocyte competence, reflected by pregnancy rates (17–19). However, while the pre-ovulatory follicles reflect the response to OS, the obtained oocytes are the concrete result of the complete procedure (stimulation and oocyte retrieval). In fact, only mature oocytes (MII) are used in IVF laboratories to obtain embryos with an implantation potential.

Further, Alviggi et al. proposed to combine the FORT index to follicle-to-oocyte (FOI) index defined by the ratio between the total number of oocytes collected and the number of antral follicles (oocyte number/antral follicle Count X100) (20). According to this publication, a normal FOI should be >50%. However, no data has shown if FOI index was correlated with pregnancy outcomes. Considering this, we investigated whether a different FOI index might correlate with IVF success rates and thus might be a more appropriate indicator of ART outcome.

The aim of our study was to evaluate the predictive value of new ovarian stimulation indicators on implantation (IR) and live birth (LB) rates. For this purpose, we decided to calculate the FOI ratio defined by the number of retrieved oocytes divided by the AFC score (Oc/AFC). To avoid the potential bias due to inter-operator variability during OS monitoring and oocyte retrieval (21), we defined the same ratios but based on the total number of mature oocytes (MII) this time (MII/AFC). Secondarily, we investigated whether the same previous ratios but using serum AMH level as denominator (Oc/AMH and MII/AMH) would be predictive of ART success.

## Materials and Methods

### Study Population

We conducted a retrospective study using an anonymized IVF database, containing clinical and laboratory parameters of the Foch Hospital ART Center in Suresnes, France. All first IVF cycles carried out in our center from September 2016 to December 2017 of women aged between 18 and 43 years were potentially included. The final inclusion was possible in the absence of the following exclusion criteria: absence of one of the two ovaries, previous attempt in another center, fertilization failure, cycles with *in vitro* maturation and cycles with non-transferred frozen embryos without having obtained a LB after previous embryo transfer (ET) from the same IVF cycle.

### Ethical Approval

All patients have signed an informed consent, allowing the use of their medical records for research purposes, as long as the patient's identity is protected, and data analysis is anonymized. This study was performed under retrospective protocol IRB number: 00012437 approved by the Foch hospital ethical committee.

### Patient Characteristics Measurements

#### AMH and AFC Measurements

We collected only AMH and AFC values measured within 12 months prior to the ovarian stimulation cycle. They were obtained at time of initial patient screening during the first 3 days of the menstrual cycle. AMH value were determined by an automated multi-analysis system using a chemiluminescence technique (Architect, Abbott, Les Clayes-sous-Bois, France) or by an immune-enzymology technique (EIA Immunotech, Beckman-Coulter, France).

To determine AFC, the ovarian ultrasound scans were performed also on the first 3 days of the menstrual cycle using a 5.0–9.0 MHz multifrequency transvaginal probe (Voluson™ S10 system, GE Healthcare). We determined, at baseline, the number of all follicles measuring 2–9 mm in diameter in both ovaries.

### Patient's Treatment

#### Ovarian Stimulation Protocol

Starting on the 20th day of previous menstrual cycle, the patients received estrogen pill (Provames® 2 mg, Sanofi Aventis, France) in an attempt to obtain a uniform follicular cohort. ART treatment followed our routine protocols. Ovarian stimulation was achieved using highly purified urinary gonadotropins: recombinant FSH, urinary FSH and/or menotropins. Individually set doses of hormones were used, ranging from 150 to 600 IU of FSH per day using an antagonist protocol. Development of ovarian follicles was monitored by transvaginal ultrasonography (TVUS) starting on the 6th day of OS. If necessary, hormonal doses were adjusted to generate an optimal response. Daily antagonist was systematically introduced from the 6th day of OS onwards, in order to inhibit ovulation of growing follicles. During the last days of OS, patients had daily visits at our institution for TVUS and hormonal examinations in order to identify the proper timing for triggering. Final oocyte maturation was typically induced when ≥3 pre-ovulatory follicles (16–22 mm in diameter) were observed and E2 levels per pre-ovulatory follicle were >200 pg/ml. This was done using a GnRH-agonist, triptorelin at the dose of 0.2–0.3 mg (Decapeptyl®, Ibsen Pharmaceuticals, France) if there was a risk of ovarian hyper stimulation syndrome OHSS, or at dose of 0.2 mg combined with 0.25 mg of choriogonadotropin alfa (Ovitrelle®. Merck Pharmaceuticals, France) if the risk was low.

Oocyte retrieval was performed 37 h after triggering. Fertilization was achieved with either intracytoplasmic sperm injection (ICSI) or classic *in vitro* fertilization (IVFc) depending on sperm parameters. When using IVFc, oocyte maturation was assessed after denudation at day 1 after fertilization. Oocytes with one polar body before ICSI and one or two polar bodies on day 1 after IVFc were considered mature (MII).

Fresh embryo transfers (ET) were planned on day 3 or day 5 according to the medical indication. However, embryo freezing (for supernumerary embryos or as part of the differed freeze-all strategy) was always performed at blastocyst stage on day 5 or day 6. Fresh and frozen blastocyst transfers were performed if blastocyst reached a full blastocyst expansion degree with inner cell mass and trophectoderm cells compatible with a transfer (22). No PGT-A was performed.

#### Frozen Embryo Transfer

Endometrial preparation was achieved with a priming phase using oral E2, as follows: 4 mg/day from day 1 to 4; 6 mg/day from day 4 to 9; 8 mg/day from day 9 onwards. Endometrial thickness was monitored by TVUS, while serum E2 and progesterone were assessed in order to rule out premature ovulation prior to initiation of progesterone supplementation. Thereafter, progesterone was added using vaginal capsules (Utrogestan®, Besin Pharma, France) at the dose of 200 mg and subcutaneous injections of progesterone (Progiron®, IBSA, France) at the dose of 25 mg/day. Warmed blastocysts were transferred on the 6th day of progesterone treatment. Hormonal treatment was pursued until the pregnancy test and continued for 8 weeks if pregnant.

### Ovarian Yield Calculation and Primary Endpoint

For the assessment of ovarian yield four ratios were calculated in relation to the AMH and the AFC:

- First, the ovarian yield (total retrieved oocytes and in terms of mature oocytes) relative to the AFC: Oc/AFC and MII/AFC, respectively
- Second, the ovarian yield (total retrieved oocytes and in terms of mature oocytes), this time expressed in relation to each patient's AMH ng/mL level: Oc/AMH and MII/AMH, respectively.

The primary endpoint was the occurrence of live birth (LB) after the first ET and live birth rate (LBR) was defined as the number of deliveries/total number of cycles. The secondary endpoints were the occurrence of a live birth after cumulated embryo transfers (CET), the implantation rate (IR) (defined as the total number of gestational sacs/total number of transferred embryos) after the first ET and the cumulated implantation after CET.

### Statistical Analysis

Data were presented a mean with SD, or median with range, and percentage as appropriate. The Shapiro-Wilk test was performed as normality test. The association between age groups and ratios was performed using ANOVA tests.

Spearman correlations were used to evaluate the association between the implantation rate after first ET and the cumulative implantation rate with the following ratios: Oc/AMH, Oc/AFC, MII/AMH, and MII/AFC and to evaluate the correlation between AMH and AFC.

Finally, linear regression models were used to quantify the relationship between implantation rates and the different ratios. A multivariate model incorporating age as a confounding factor followed the univariate linear model.

To measure the association of the different ratios with the presence of a LB (after the first ET and cumulatively), Student tests were performed, followed by a univariate and then a multivariate logistic regression model to account for age for each ratio tested.

The tests were bilateral, the significance level set at 5% and the analyses were performed using STATA software (Statacorp L, Texas, USA).

## Results

### Characteristics of Patients

A total of 264 IVF cycles were included in the study. Mean age in the study cohort was 35.2 ± 4.0 years. A total of 144 patients (54.5%) were under 36 old years (yo), 67 (25.4%) between 36 and 39 yo and 56 (20.1%) over 39 yo. Medical indications for ART were male factor, tubal factor, endometriosis, polycystic ovary syndrome (PCOS) and idiopathic infertility. In 62% of cases, couples presented simultaneously more than one medical indications. Mean AMH was 2.68 ± 2.65 ng/ml while mean AFC was 18.0 ± 11.2.

Out of 264 IVF cycles, 185 resulted in a fresh ET (70.1%) and 57 (21.6%) were intended to freeze-all strategy. For 22 cycles (8.3%) no embryo for transfer or freezing was obtained.

After the first ET (fresh or frozen), 103 women obtained a clinical pregnancy, among those, 17 experienced spontaneous pregnancy loss, and 86 delivered a healthy baby. The IR was 31 ± 39% and the LBR was 32.6%. After CET, we obtained 157 clinical pregnancies and 119 live births, cumulated IR and LBR were 35 ± 38 and 45.1%, respectively.

The average Oc/AFC and MII/AFC ratios were 6.6 ± 4.7 and 5.3 ± 4.0, respectively. The average Oc/AMH and MII/AMH ratios were 0.7 ± 0.4 and 0.6 ± 0.3, respectively. There was no significant difference in these four ratios according to the age categories of women. Although for the Oc/AMH ratio, we observed a tendency but not significant (*P* = 0.05) to an increase in the ratio in patients over 39 years of age (Table 1).

**Table 1:** Oocyte yield indices in infertile women with or without live birth after the first embryo transfer.

	**Live birth**		
	**Yes**	**No**	
**Ratio**	**Mean ± SD**	**Mean ± SD**	***P*-value**
Oc/AFC	0.82 ± 0.4	0.71 ± 0.4	**0.03**
MII/AFC	0.68 ± 0.3	0.57 ± 0.4	**0.02**
Oc/AMH	6.5 ± 5.1	6.8 ± 3.9	0.52
MII/AMH	5.57 ± 3.3	5.19 ± 4.3	0.42

*Data are presented as mean with standard deviation*.

*Oc/AFC, total retrieved oocytes relative to antral follicular count*.

*MII/AFC, total mature oocytes relative to antral follicular count*.

*Oc/AMH, total retrieved oocytes relative to serum AMH ng/mL level*.

*MII/AMH, total mature oocytes relative to serum AMH ng/mL level*.

*Bold values for statistically significant differences*.

In our population, we observed a strong positive correlation between AMH and AFC: when one increased, so did the other (*P* < 0.001).

### Patient's Results

In an effort to determine whether Oc/AFC, MII/AFC, Oc/AMH, and MII/AMH ratio had an impact on the occurrence of LB and on IR in association with female age, we examined these 4 ratios using a bivariate age-grouping paradigm. The dataset was stratified according to female age, studying the oocyte yield in the following age groups: <36, [36–39], >39 years.

#### The Occurrence of Live Birth

When comparing the average of cycle's ratios according to the presence or absence of a LB after the first ET, there was a significantly higher average of Oc/AFC and MII/AFC for LB group (0.82 ± 0.4 vs. 0.71 ± 0.35 and 0.57 ± 0.4 vs. 0.68 ± 0.3, respectively, *P* < 0.05) (Table 1).

Increased Oc/AFC and MII/AFC ratios were associated with higher number of LB. Furthermore, in the age-adjusted multivariate analysis, only the MII/AFC ratio remained significant (*P* = 0.04), in contrast to the Oc/AFC ratio which had a limit *P* of 0.05 (Table 2).

**Table 2:** Odds ratios of oocyte yield indices after first embryo transfer according to age groups Naissance.

	**Logistic regression**			**Logistic regression age-adjusted**		
	**OR**	**IC95%**	***p***	**OR**	**IC95%**	***p***
**Oc/AFC**	**2.14**	**[1.08–4.21]**	**0.03**	**2.00**	**[0.99–4.01]**	**0.05**
Age (years)						<0.01
<36				1		
[36–39]				0.55	[0.3–0.99]	
>39				0.23	[0.08–0.62]	
**MII/AFC**	**2.50**	**[1.17–5.37]**	**0.02**	**2.28**	**[1.05–5.00]**	**0.04**
Age (years)						<0.01
<36				1	–	
[36–39]				0.55	[0.30–0.99]	
>39				0.23	[0.08–0.63]	
**Oc/AMH**	**1.01**	**[0.96–1.07]**	**0.56**	**1.03**	**[0.98–1.09]**	**0.24**
Age (years)						<0.01
<36				1	–	
[36–39]				0.51	[0.28–0.92]	
>39				0.20	[0.07–0.57]	
**MII/AMH**	**1.02**	**[0.97–1.09]**	**0.47**	**1.04**	**[0.97–1.11]**	**0.22**
Age (years)						<0.01
<36				1	–	
[36–39]				0.51	[0.28–0.93]	
>39				0.21	[0.07–0.57]	

*Oc/AFC, total retrieved oocytes relative to antral follicular count*.

*MII/AFC, total mature oocytes relative to antral follicular count*.

*Oc/AMH, total retrieved oocytes relative to serum AMH ng/mL level*.

*MII/AMH, total mature oocytes relative to serum AMH ng/mL level*.

However, the Oc/AMH and MII/AMH ratios had no effect on the occurrence of LB at either age grouping as shown in Tables 1, 2.

When comparing the average of cycle's ratios according to the presence or absence of a LB after CET, there was a significantly higher average of MII/AFC for LB group (0.66 ± 0.3 vs. 0.56 ± 0.30, *P* = 0.02) (Table 3).

**Table 3:** Oocyte yield indices in infertile women with or without live birth after the cumulative embryo transfer.

	**Live birth**		
	**Yes**	**No**	
**Ratio**	**Mean ± SD**	**Mean ± SD**	***P*-value**
Oc/AFC	0.79 ± 0.4	0.72 ± 0.4	0.14
MII/AFC	0.66 ± 0.3	0.56 ± 0.3	**0.02**
Oc/AMH	6.54 ± 3.8	6.65 ± 5.4	0.84
MII/AMH	5.43 ± 3.2	5.21 ± 4.6	0.65

*Data are presented as mean with standard deviation*.

*Oc/AFC, total retrieved oocytes relative to antral follicular count*.

*MII/AFC, total mature oocytes relative to antral follicular count*.

*Oc/AMH, total retrieved oocytes relative to serum AMH ng/mL level*.

*MII/AMH, total mature oocytes relative to serum AMH ng/mL level*.

*Bold values for statistically significant differences*.

Spearman's correlation analysis showed no association between Oc/AFC, Oc/AMH, and MII/AMH ratios and the occurrence of LB after cumulative embryo transfer. However, an increase in the MII/AFC ratio raised LB occurrence chances (*P* = 0.02). Which was confirmed after age-adjusted multivariate analysis (*P* = 0.04) (Table 4).

**Table 4:** Odds ratios of oocyte yield indices after cumulative embryo transfer according to age groups.

	**Logistic regression**			**Logistic regression age-adjusted**		
	**OR**	**IC95%**	***p***	**OR**	**IC95%**	***p***
**Oc/AFC**	**1.63**	**[0.85–3.14]**	**0.14**	**1.52**	**[0.77–2.99]**	**0.22**
Age (years)						<0.01
<36				1		
[36–39]				0.49	[0.28–0.86]	
>39				0.22	[0.09–0.52]	
**MII/AFC**	**2.42**	**[1.14–5.14]**	**0.02**	**2.23**	**[1.02–4.88]**	**0.04**
Age (years)						<0.01
<36				1	–	
[36–39]				0.50	[0.29–0.87]	
>39				0.23	[0.10–0.53]	
**Oc/AMH**	**0.99**	**[0.94–1.05]**	**0.85**	**1.01**	**[0.96–1.07]**	**0.69**
Age (years)						<0.01
<36				1	–	
[36–39]				0.47	[0.27–0.82]	
>39				0.21	[0.09–0.51]	
**MII/AMH**	**1.01**	**[0.95–1.08]**	**0.66**	**1.03**	**[0.97–1.10]**	**0.33**
Age (years)						<0.01
<36				1	–	
[36–39]				0.47	[0.27–0.82]	
>39				0.21	[0.09–0.49]	

*Oc/AFC, total retrieved oocytes relative to antral follicular count*.

*MII/AFC, total mature oocytes relative to antral follicular count*.

*Oc/AMH, total retrieved oocytes relative to serum AMH ng/mL level*.

*MII/AMH, total mature oocytes relative to serum AMH ng/mL level*.

#### The Implantation Rate

There was an association between the Oc/AFC ratio and IR, and between MII/AFC ratio and the IR after the first ET: the increase in those ratios favored the increase in the IR after the first ET (OR = 1.16 IC95% [1.02–1.32], *P* = 0.02 and OR = 1.23 IC95% [1.06–1.42], *P* < 0.01, respectively). This association was found in the age-adjusted multivariate model (OR = 1.14 IC95% [1.01–1.30], *P* = 0.04 and OR = 1.20 IC95% [1.04– 1.39], *P* = 0.01). However, according to our results the Oc/AMH and MII/AMH ratios had no effect on IR at either age grouping.

Spearman's correlation analysis showed no association between Oc/AFC, Oc/AMH, and MII/AMH ratios and IR after cumulative embryo transfer. However, an increase in the MII/AFC ratio raised IR (ρ = 0.15, *P* = 0.02), but after age-adjusted multivariate analysis, this impact became not significant.

## Discussion

Our study aimed to evaluate whether the calculation of an ovarian yield according to the number of retrieved oocytes or mature oocytes, previously described to assess ovarian response, was predictive of ART success. As oocyte potential may vary according to these ratios, we chose to indirectly estimate this oocyte potential by evaluating implantation rate and live birth rate after embryo transfer. Moreover, since the embryo with the best potential within a cohort is transferred first, we chose to evaluate results after the first ET as the primary endpoint. We also chose to assess cumulative IR and LBR which provide additional information but potentially favor patients with high AFC, such as PCOS who will have statistically more supernumerary frozen embryos (8).

The advantage of those index compared to the FORT index described above is that they evaluate both the response to OS and the technical process of oocyte retrieval and recovery. It is interesting to note that FORT and MII/Oc are predictive of success in ART treatment while they are not quite comparable but possibly complementary. In fact, in the calculation of the FORT, the pre-ovulatory follicles of intermediate size (14–16 mm) are not taken into account whereas they can give mature oocytes, taken into account by our index (23).

Furthermore, while the FORT is evaluated during stimulation, and could have an impact on the decision to cancel a cycle or not, our index can only be estimated once the oocyte retrieval has been carried out, but it carries the advantage of being estimated in a more objective way (number of oocytes and number of mature oocytes) in relation to the FORT. The proposed index in this study avoids the subjectivity of ultrasound in the evaluation of the number of pre-ovulatory follicles when these are numerous, or when imaging is difficult and depends on the operator. Therefore, our proposed index could be an additional and complementary criterion to the FORT allowing to better adjust the treatment during a possible second cycle in case of failure.

As expected, we found a very strong correlation between AMH and AFC in patients. Moreover, the age of the patients had the greatest impact on implantation and birth rates (24).

The Oc/AFC ratio had a significant association with the IR, and at the limit of significance with the presence of a LB after the first ET, whatever the age of the patient. On the other hand, on the cumulative results, no association was found for this ratio. The MII/AFC ratio proved to be more influential, with a strong association with ART outcomes after first ET and a strong association on LB after cumulative transfers. These two ratios seem promising for assessing the quality of an ovarian response to gonadotropins. The fact that the ovarian yield in terms of mature oocytes is more significantly predictive than that of total oocytes is consistent with the relevance of the first one because it does not consider inter-operator variability during the oocyte retrieval procedure, and represents the expected ultimate outcome for OS, namely; obtaining mature oocytes.

In addition, MII/AFC can be a good indicator of quality in an ART center. Pirtea et al. found that the number of oocytes retrieved compared to the expected retrieved could not be used as a good key performance indicator because of its high variability (25). On the other hand, it seems to us that the number of MII can be used to estimate the effectiveness of an ART center in terms of OS protocols and oocyte retrieval efficacy.

Furthermore, in our study, no ratio using AMH in its denominator was predictive. The fact that the numerators and denominators of those ratios do not have the same units (number and ng/mL) may make them more complex and their correlation uncertain. Thus, a patient with an AMH of 0.2 ng/mL who obtains 2 MII at retrieval will have a ratio of 10, and a patient with an AMH of 1 ng/mL who obtains 10 MII will also have a ratio of 10, although we can assume that their prognosis is not the same.

In addition, the lack of significance may be due to the lack of standardization in AMH dosing with different techniques, making its relevance uncertain, or to the complex role of AMH, which also inhibit the development of pre-antral follicles in response to FSH (26), making it inversely correlated to the FORT (16).

Our data suffer from the weakness of retrospective nature of its design and the lack of homogeneity and AMH dosing techniques making the interpretation of ratios involving it uninterpretable. However, it has the advantage of including a population that reflects our day-to-day practices without excluding PCOS profiles and considering only first attempts in our center, contrary to what was previously done. The outcomes of ART attempts were evaluated up to LB and we were able to compare the cumulative results, which is not often done in previous studies (15, 26).

In conclusion, the search for the ideal index capable of predicting oocyte potential and the chances of obtaining a live birth remains extremely relevant. Our indexes involving serum AMH level have not demonstrated a sufficiently strong prediction. On the other hand, our indices involving AFC (Oc/AFC and MII/AFC) seem promising and could be interesting candidates to validate in a prospective cohort study.

## Data Availability Statement

The raw data supporting the conclusions of this article will be made available by the authors, without undue reservation.

## Author Contributions

MP, RY, AB, and JA contributed to the protocol elaboration, data collection, results interpretation, and manuscript writing and editing. PP and DZ contribute to results analysis and manuscript editing. JT contribute to statistical analysis. All authors contributed to the article and approved the submitted version.

## Conflict of Interest

The authors declare that the research was conducted in the absence of any commercial or financial relationships that could be construed as a potential conflict of interest.

## Publisher's Note

All claims expressed in this article are solely those of the authors and do not necessarily represent those of their affiliated organizations, or those of the publisher, the editors and the reviewers. Any product that may be evaluated in this article, or claim that may be made by its manufacturer, is not guaranteed or endorsed by the publisher.
